# Internet Access and Use in Adults With Hearing Loss

**DOI:** 10.2196/jmir.2221

**Published:** 2013-05-09

**Authors:** Elisabet Sundewall Thorén, Marie Öberg, Gunilla Wänström, Gerhard Andersson, Thomas Lunner

**Affiliations:** ^1^Division of Technical AudiologyDepartment of Clinical and Experimental MedicineLinköping UniversityLinköpingSweden; ^2^Eriksholm Research CentreOticon A/SSnekkerstenDenmark; ^3^Hearing ClinicCounty Council of ÖstergötlandLinköpingSweden; ^4^The Swedish Institute for Disability ResearchLinköping UniversityLinköpingSweden; ^5^Department of Behavioural Sciences and LearningLinköping UniversityLinköpingSweden; ^6^Department of Clinical NeuroscienceKarolinska InstitutetStockholmSweden

**Keywords:** hearing loss, hearing rehabilitation, Internet, trends

## Abstract

**Background:**

The future rehabilitation of adults with hearing loss is likely to involve online tools used by individuals at home. Online tools could also be useful for people who are not seeking professional help for their hearing problems. Hearing impairment is a disability that increases with age, and increased age is still associated with reduced use of the Internet. Therefore, to continue the research on online audiological rehabilitative tools for people with hearing loss, it is important to determine if and to what extent adults with hearing loss use the Internet.

**Objective:**

To evaluate the use of the Internet and email in a group of adults with hearing loss and to investigate if their use of Internet and email differed between genders, among different age groups, and how it compared with the general population in Sweden.

**Methods:**

Questionnaires containing multiple-choice questions about Internet access, email use, and educational level were mailed to individuals with hearing loss, who were registered as patients at a hearing aid clinic. Out of the 269 invited participants, 158 returned a completed questionnaire, which was a response rate of 58.7%.

**Results:**

The results showed that 60% (94/158) of the participants with hearing loss used computers and the Internet. The degree of hearing loss in the group of participants did not explain the level of Internet usage, while factors of age, gender, and education did (*P*<.001). More men than women used the Internet (OR 2.54, 95% CI 1.32-4.91, *P*<.001). Use of the Internet was higher in the youngest age group (25-64 years) compared to the oldest age group (75-96 years, *P*=.001). A higher usage of the Internet was observed in the participants with hearing loss, especially the elderly, when compared with the general population of Sweden (OR 1.74, 95% CI 1.23-3.17, *P*=.04).

**Conclusions:**

We conclude that the use of computers and the Internet overall is at least at the same level for people with hearing loss as for the general age-matched population in Sweden, but that this use is even higher in specific age groups. These results are important for the future work in developing and evaluating rehabilitative educational online tools for adults with hearing loss.

## Introduction

The use of the Internet and communication via email, chat forums, blogs, and instant messaging have increased dramatically in western society during recent years. For groups that are suffering from different disabilities, the Internet and the use of online communication tools can be helpful because they can reach out and contact other people without being identified as disabled [1]. Research shows that people with different kinds of working disabilities benefit from using computers and the Internet, but they still represent a group with less access to these media than persons without disabilities. It has been argued that the low rate of Internet use in persons with disabilities linked to problems at work (eg, chronic pain) may be associated with socioeconomic factors and a lack of knowledge about online resources [2].

One of the most common disabilities is hearing loss, and objectively measured hearing loss often occurs prior to subjective hearing difficulties [3]. People with hearing loss describe difficulties when interacting with friends in daily communication situations. The mixing of speech with different types of background noise represents a challenging situation for hearing-impaired people. This issue is one reason why the Internet is a medium that may suit people with hearing loss and deaf people because they can use it to communicate with people with normal hearing or peers with similar problems [1,4]. The Internet can also be useful as a communication tool for people with hearing loss, eg, the use of Internet telephony, which has been shown to be particularly useful for improving speech perception [5]. Hearing impairment is a disability that increases with age [6]; usually by approximately age 50, 35-45% of adults report some kind of hearing difficulty, and it is therefore an important public health problem [7].

Higher age is typically associated with reduced use of the Internet [8]. For example, data from Europe have shown that a third of those over the age of 54 and 10% of the adults over the age of 65 use the Internet, which can be contrasted with the approximately 75% of individuals aged 16-24 years who use the Internet [9]. Furthermore, a Canadian study showed that fewer than 10% of the elderly used the Internet, with “elderly” being defined as over 65 years old [10]. An observation made in the same study was that older persons with better hearing and hearing aid users use information technology, such as the Internet, more than persons with hearing loss who were not using their prescribed hearing aids. However, the Canadian data [10] were collected in 2000, and a plausible assumption is that Internet usage has increased since then in all age groups, including the elderly [11]. For some of the people who are not using the Internet, however, it is assumed that they could benefit from available online services. Research on 86-year-olds in Sweden showed that 19% owned a computer, but only 10% were connected to the Internet. However, approximately 90% of the respondents expressed that they might benefit from the Internet if they had access to it [12]. In a study published more than 5 years ago [13], the researcher concluded that teaching the elderly how to use computers and the Internet was possible, so that, for example, the elderly could obtain information and health care contacts. In addition, including the elderly in the process of developing computers and Internet tools and adjusting the media to be more useful and attractive for many older adults were important factors [14].

The rehabilitation of people with hearing loss, audiological rehabilitation, is a complex process and not always successful. Audiological rehabilitation for people suffering from hearing loss consists of many elements, but most people with hearing loss are not offered any additional rehabilitation once their hearing aids have been fitted. In fact, a large number of adults with hearing loss are not seeking professional help to overcome their hearing problems.

Currently, approximately 67% of all Internet users use it to search for online health-related information [15]. In a study from the United Kingdom, Henshaw et al concluded that the Internet is a useful medium for offering hearing health care, especially to the adults with hearing loss who are typically not seeking hearing health care [16]. By using the Internet during audiological rehabilitation, relevant elements could be included in the rehabilitation without the inconvenience of traveling to a hearing health center away from home. In the near future, parts of the rehabilitation and contact with the professionals could be expected to occur via the Internet [17,18]. Therefore, to continue the research on online audiological rehabilitative tools for people with hearing loss, it is important to determine if and to what extent adults with hearing loss use the Internet.

The objective of this study was to evaluate the use of the Internet, computers, and email in a group of adults with hearing loss. We also investigated whether the use of the Internet, computers, and email differed between genders and different age groups and to what degree age, gender, education, and hearing loss could explain the amount of Internet usage. The final aim of our study was to investigate whether there was a difference in the use of the Internet, computers, and email between the general population of Sweden [19] and a group of adults with hearing loss.

## Methods

### Recruitment and Procedure

In this study, we used systematic sampling by inviting a selected group of hearing aid users from the University Hospital in Linköping, Sweden. Every fourth person who had finished hearing-aid rehabilitation at the University Hospital during 2008 and who did not meet our exclusion criteria was asked to participate in the study via invitation letters sent by regular mail. The defined exclusion criteria were if the potential participant was unable to communicate in Swedish (ie, used an interpreter during the hospital visit) or was under the age of 18 years. During 2009, invitation letters were sent by mail to a total of 269 individuals. The average age of the invited participants was 73.4 years (range 20-98 years; SD 13.3). The invited participants included 154 out of 269 men (57.2%) and 115 out of 269 women (42.8%).

### Study Participants

Among the invited participants, 173 out of 269 individuals returned their questionnaires (response rate 64.3%), and 158 out of 269 (58.7%) returned a completed questionnaire. We did not send out reminders to the invited participants. The age of the participants who returned a complete questionnaire ranged from 31-96 years (mean 73.6 years; SD 12.2 years). Of the included participants, 96 out of 158 were men (60%) and 62 out of 158 were women (40%). The majority, 85 out of 158 participants (54%), had completed 9 years of elementary school. The lowest number of individuals, 31 out of 158 (20%), had finished 12 years in school and 42 out of 158 (27%) had a university-level degree and therefore more than 12 years in school, as shown in Table 1. Approximately half of the participants (86/158, 54.7%) were urban citizens, living in cities with more than 100,000 residents. The remainder of the participants lived in smaller villages in the countryside. In total, 110 out of 269 (40.9%) invited individuals were counted as nonrespondents because they either did not return the questionnaire or returned an incomplete questionnaire. The mean age of the nonrespondents was 73.2 years (range 20-98, SD 14.9 years). There was no significant difference due to age between the participants who returned a complete questionnaire and those who did not return the questionnaire (*P*=.39).

### Hearing Loss

**Figure 1:**
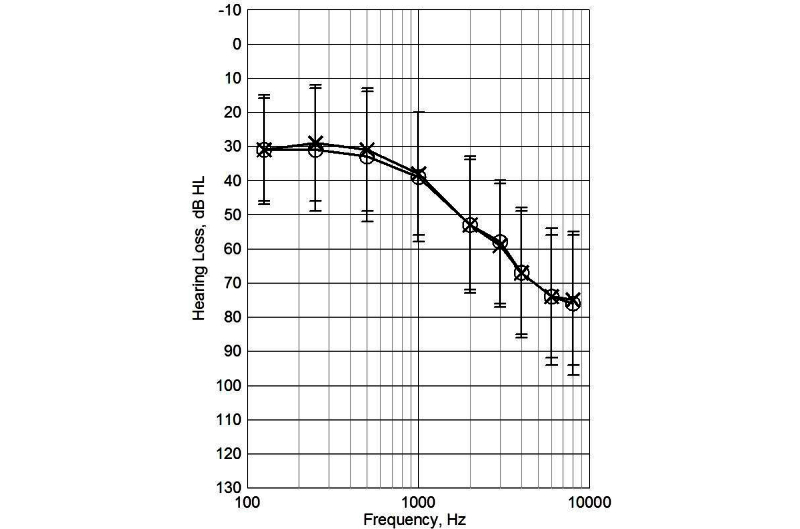
Average (SD) hearing loss of the participants (dB HL; right ear points marked with circles and left ear with crosses).

Measurements of the participants’ hearing losses, as measured by the pure-tone air-conducted hearing thresholds (ISO 8253-1 1989) were collected from their latest visit to the hearing clinic. The pure tone average of the better ear at the four frequencies of 500, 1000, 2000, and 4000 Hz was, on average, 40 dB HL (SD 15 dB HL; Figure 1). There was a significant correlation between age and the degree of hearing loss (*r*=-0.46, *P*<.001), indicating that the degree of hearing loss increased with increasing age, as expected.

### Outcomes

A questionnaire containing multiple-choice questions about Internet access, email use, and educational level was mailed to the invited participants. The questionnaire was sent together with a letter and a pre-paid reply envelope. The questionnaire contained the following questions: (1) Do you have a computer at home?, (2) Do you have access to a computer outside your home?, (3) Do you have access to the Internet?, (4) Do you search for information via the Internet?, (5) How often do you use the Internet?, (6) Do you have an email address?, (7) Do you use your email address?, (8) How often do you use your email address?, and (9) What kind of education do you have? (see Multimedia Appendix 1).

The medical ethical committee in Linköping, Sweden, approved the protocol.

### Statistical Analysis

To statistically test the aims of the study, descriptive analysis, Pearson’s chi-square test (χ^2^), and multiple regression analysis were performed. For all analyses, an alpha level of <.05 was used as statistical significance. A multiple regression analysis was used to investigate whether age, education, gender, or hearing loss could predict Internet use and to investigate how much of the variance in Internet use could be explained by age, education, gender, and hearing loss, as shown. The statistical software package Statistica 10, StatSoft, was used.

## Results

### Questionnaires

We were interested in the distribution of use of the Internet, computers, and email in a group of adults with hearing loss. The results are presented in Table 1 for the whole group, for three different age groups, and for men and women in the different age groups. We found that 60% (94/158) of respondents had a computer at home. Out of those who had a computer at home, only 1 participant did not have access to the Internet. Furthermore, out of the participants who used the Internet, a clear majority of 83% (77/93) answered that they used the Internet monthly or more often. Half of the participants (81/158, 51%) had an email address, and all except 7 participants used it daily or multiple days per week.

The second aim was to investigate whether Internet, computer, and email use differed between genders. The results showed that significantly more men than women had a computer (*P*<.001), had access to the Internet (*P*=.01,) and used email (*P*<.001), as shown in Table 2. The results are in agreement with general Internet use in the Swedish population [19], showing significantly higher usage of computers and the Internet by men than women (*P*<.001).

The third aim of the study was to analyze the data with respect to age groups to investigate if Internet use and email use were significantly lower in the elderly compared with the younger participants. We divided the participants into three age groups, as shown in Table 3. The age groups were defined so that the results could be compared with data on Internet use in the general population of Sweden and with respect to the typical correlation between hearing impairment and a gradual acceleration in the age of retirement (65 years). The results showed that there were significantly more participants in the younger age groups that had access to a computer than in the older group (*P*<.001), as shown in Table 3. Similar results were seen with respect to the use of Internet and email, meaning that the use of these services was significantly more common in the younger groups than in the older group (*P*<.001).

Multiple logistic regression analysis was done to investigate whether age, education, gender, or hearing loss could predict Internet use. Multiple logistic regressions were also used to investigate how much of the variance in Internet use could be explained by age, education, gender, and hearing loss, as seen in Table 4. As shown in Table 4, the partial correlations were significant for all factors except for hearing loss. Therefore, the factor of hearing loss was excluded in further analysis. About 14% of the variance in Internet use was explained by age, around 12% was explained by education, and less than 3% was explained by the gender of the participants.

The final aim was to compare the data generated in this study with the data from a typical Swedish population [18] in order to investigate if the group of adults with hearing loss used information technology more than their peers in the general population, as shown in Table 5.

Additionally, each age group from the two datasets was compared, as shown in Table 3. In the youngest age group I (25-64 yrs), we did not find any significant differences in Internet use (*P*=.06) or computer access at home (*P*=.10). In age group II (65-74 yrs), there were no significant differences in computer access at home when comparing the two datasets (*P*=.30), but the participants from the group of adults with hearing loss had significantly more access to Internet than the people in the general population (*P*=.05). In age group III (75-96 yrs), we found significant differences between the two datasets in both computer access (*P=*.004) and Internet use (*P*=.02), which were more common in the group of participants with hearing loss.

**Table 1:** The collected data (%) on the use of Internet, computers, and email overall and in the different age groups.

					Education		
		Computer	Internet	Email	9 yr	12 yr	>15 yr
**Total**							
	All (N=158)	59.5	58.9	51.2	50	18.3	24.1
	Men (N=96)	70	67.7	63.5	51	19.8	21.9
	Women (N=62)	43.5	45.2	30.1	48.4	16.1	27.4
**Age 25-64**							
	All (N=31)	100	100	87.1	25.8	35.5	38.7
	Men (N=20)	100	100	90	25	35	40
	Women (N=11)	100	100	81.8	27.3	36.4	36.4
**Age 65-74**							
	All (N=41)	75.6	78	65.8	48.8	24.4	26.8
	Men (N=28)	82.1	82.1	78.6	50	25	25
	Women (N=13)	61.5	69.2	38.5	46.2	23.1	30.8
**Age 75-96**							
	All (N=86)	37.2	34.9	30.2	59.3	9.3	17.4
	Men (N=48)	50	45.8	43.8	62.5	10.4	14.6
	Women (N=38)	21.1	21.1	13.2	55.3	7.9	21.1

**Table 2:** Results from the chi-square tests (χ^2^) and odds ratio (OR) comparing gender and comparing with the dataset from the general population of Sweden (data on email use not collected in the dataset from the general population).

		χ^2^	*P* value	OR	Lower 95% CI	Upper 95% CI
**Current data (n=158)**						
	PC use	10.77	.001	3.03	1.56	5.90
	Internet use	11.52	<.001	2.54	1.32	4.91
	Email use	16.31	<.001	4.04	2.04	8.00
**General population (n=6292)**						
	PC use	47.09	<.001	1.53	1.35	1.73
	Internet use	35.34	<.001	1.42	1.27	1.59
	Email use	—	—	—	—	—

**Table 3:** Results from the chi-square tests (χ^2^) and odds ratio (OR) comparing age groups (AG) and with the dataset from the general population of Sweden (data on email use not collected in the dataset from the general population).

		AG II					AG III					General population				
		χ^2^	OR	Lower, 95% CI	Upper, 95% CI	*P*	χ^2^	OR	Lower, 95% CI	Upper, 95% CI	*P*	χ^2^	OR	Lower, 95% CI	Upper, 95% CI	*P*
**AG I**																
	Computer	8.78	^a^	^a^	^a^	.003	38.72	^a^	^a^	^a^	<.001	2.77	^a^	^a^	^a^	.10
	Internet	7.78	^a^	^a^	^a^	.01	36.15	^a^	^a^	^a^	<.001	3.63	^a^	^a^	^a^	.06
	Email	4.25	3.50	1.02	12.01	.04	29.74	15.58	4.95	49.02	<.001	—	—	—	—	—
**AG II**																
	Computer						18.52	5.79	2.50	13.40	<.001	1.12	1.48	0.71	3.06	.29
	Internet						18.45	6.00	2.54	14.17	<.001	3.95	2.11	0.99	4.48	.05
	Email						14.49	4.45	2.01	9.83	<.001	—	—	—	—	—
**AG III**																
	Computer											8.29	1.98	1.23	3.17	.004
	Internet											5.62	1.74	1.10	2.77	.02
	Email											—	—		—	—

^a^The value could not be calculated due to few data points in the cell.

**Table 4:** Multiple logistic regression results for prediction of Internet use.

	b^a^	b	*P* value
Intercept		30.88	<.001
Age	-0.38	-0.02	<.001
Education	-0.34	0.07	<.001
Gender	0.17	0.18	<.01
Hearing loss	0.03	0.07	.64

^a^b=raw coefficients in the multiple regression equation.

**Table 5:** The use of computers and the Internet (in %) in the general population of Sweden.

		Computer	Internet
**Total**			
	All (N=6292)	79.7	76.8
	Men (N=3219)	83.2	80.0
	Women (N=3372)	76.4	73.8
**Age group I**			
	All (N=4829)	91.8	89.4
	Men (N=2441)	92.1	89.5
	Women (N=2388)	91.5	89.4
**Age group II**			
	All (N=886)	67.7	62.7
	Men (N=425)	72.7	68
	Women (N=460)	63.3	58
**Age group III**			
	All (N=878)	25.5	21.3
	Men (N=354)	34.2	28.8
	Women (N=524)	19.3	16.2

## Discussion

### Principal Findings

The aim of this study was to evaluate the use of the Internet, computers, and email in a group of adults with hearing loss. Furthermore, we investigated if the use of Internet, computers, and email differed between genders and different age groups. The results relating to our first objective indicate that the use of Internet and email is high in the total group of adults with hearing loss, even though it is significantly more common in the younger age groups. We interpret our results of computer and Internet use as valid since the participants reported use of email was nearly the same as computer and Internet use, and we therefore expect that the participants in our sample are using both their computers and their Internet connections. These findings were expected based on the results from earlier studies showing that Internet use in the elderly population is low [8,12]. However, Internet use in adults with hearing loss has barely been described in the literature [16].

In this study, we also identified a difference due to gender. Our results showed that it was more common for men than women to use computers, the Internet, and email. These findings were expected in this group of participants due to prior research evaluating how gender differences correlate with Internet usage [16]. Our results are also in line with findings from the general Swedish population, which show a significant difference in the use of the Internet and computers between genders [19].

Because hearing loss increases with age, the final objective in our study was to evaluate the pattern of Internet use among the elderly and compare with data from the general population of Sweden. More specifically, our aim was to evaluate whether we could detect a higher rate of Internet use among adults with hearing loss. In summary, the results from the multiple regression analysis indicate that Internet use in a group of adults with hearing loss can be explained by age, education, and gender. More specifically, the highest usage of the Internet is seen among the youngest age group and males with higher education levels, and the lowest usage is among the elderly and women with less education. These results are consistent with newly reported findings from Henshaw et al [16] who investigated the association between age, socioeconomic status, and gender due to self-reported usage of computers and the Internet. The findings are also in line with reports of general Internet use in the Swedish population [15] showing that age, gender, and education still have a strong association with Internet use, especially among the elderly.

When comparing our data with information technology use in the general Swedish population [19], we found that individuals with hearing loss were more likely to use computers and have Internet access than individuals with normal hearing. Interestingly, a comparison of our data in elderly people with hearing loss with data from Europe [9] showed that approximately 10% of the elderly above 65 years use the Internet, while in our study, 50% of those over 65 use computers and the Internet. Recently reported data show that Sweden represents one of the top five countries with high access and Internet usage [15]. This could explain why our study shows a reasonably higher use of the Internet by elderly persons with hearing loss than a comparable study from United Kingdom [16].

A reasonable overall interpretation of our findings, together with previous findings, is that people with decreasing hearing sensitivity are using computers and the Internet and also that these media can be useful tools to help the elderly with hearing loss get access to rehabilitation from home [2,16,18].

### Limitations

In this study, we reported on the use of the Internet, computers, and email in a sample from Sweden, but usage in a similar group in other parts of Europe might be different. The data presented in the study can be seen as a reflection of the present, but the reported use of this type of information technology typically continues to increase over time, especially in elderly people [11]. However, we find the data to be representative as a sample from the population of people with hearing loss recruited at a hearing clinic in the county of Östergötland because all age groups are represented and because we could see the expected increase of hearing loss with increased age. Further analyses of the Internet, computer, and email usage in different groups of individuals with hearing loss and of different ages should involve more participants to investigate if our findings apply to the general population of people with hearing loss. We had a response rate of 59%, without any missing data in the questionnaires, which we consider rather good since the majority of our participants were elderly. Questionnaires mailed to elderly people are not always recommended as the first choice because a high rate of missing data and a lack of willingness to fill out questionnaires have been reported [20]. Determining the use of the Internet, computers, and email in the group of nonrespondents could, however, be interesting.

### Future Research

This study was conducted to evaluate whether information technology was used by people with hearing loss in order to explore if developing online rehabilitation tools for them would be fruitful. By using the Internet in the rehabilitation of adults with hearing loss, they could be informed and guided about communication strategies, hearing tactics, ways to handle hearing aids, and other issues in a cost-effective manner. Our results support the idea that, in the near future, the audiological rehabilitation process can be expected to include the Internet and that some elements of rehabilitation and contact with professionals can occur via the Internet using communication tools like email [17,18].

### Conclusion

The conclusion from this study is that, in a systematic, selective sample of hearing aid users, the use of computers, Internet, and email are overall at the same level as the general Swedish population, but this use is even higher in some specific age groups. This information is important for future work in developing and evaluating rehabilitative educational online tools for adults with hearing loss.

## Multimedia Appendix 1

Questionnaire.
