# Supplementary material for: Internet Access and Use in Adults With Hearing Loss
Source: J Med Internet Res. 2013 May 9;15(5):e91. doi: 10.2196/jmir.2221 (PMC3650922; doi:10.2196/jmir.2221)
Supplement: Supplementary file 1 [file jmir_v15i5e91_app1.pdf]

Multimedia Appendix 1. Postal questionnaire.

In this research study, we want to identify how people with hearing impairments use computers and the Internet. Below you will find nine questions and we ask you to check the box in front of the answer you agree with most.

1. **Do you have a computer at home?**

   ☐ Yes ☐ No

2. **Do you have access to a computer outside your home (e.g. at work, library etc.)?**

   ☐ Yes ☐ No

3. **Do you have access to the Internet?**

   ☐ Yes ☐ No

4. **Do you search information via the Internet?**

   ☐ Yes ☐ No

5. **If your answer was "yes" on Q4, how often do you use the Internet?**

   ☐ Daily ☐ Weekly ☐ Monthly ☐ Very seldom

6. **Do you have an email address?**

   ☐ Yes ☐ No

7. **Do you use your email address?**

   ☐ Yes ☐ No

8. **How often do you use email?**

   ☐ Daily ☐ Weekly ☐ Monthly ☐ Very seldom

9. **What is your last completed level of education?**

   ☐ Elementary School ☐ High School ☐ University
